# DNA-Damage-Induced Alternative Splicing of p53

**DOI:** 10.3390/cancers13020251

**Published:** 2021-01-12

**Authors:** Jing Chen, Dadong Zhang, Xiaodi Qin, Kouros Owzar, Jennifer J. McCann, Michael B. Kastan

**Affiliations:** 1 Department of Pharmacology and Cancer Biology, Duke University School of Medicine, Durham, NC 27710, USA; jing.chen@crownbio.com (J.C.); jennifer.mccann@duke.edu (J.J.M.); 2 Current Address-Crown Bioscience, Inc., San Diego, CA 92127, USA; 3 Duke Cancer Institute, Durham, NC 27710, USA; dadong.zhang@duke.edu (D.Z.); xiaodi.qin@duke.edu (X.Q.); kouros.owzar@duke.edu (K.O.); 4 Department of Biostatistics and Bioinformatics, Duke University Medical Center, Durham, NC 27710, USA

**Keywords:** cancer, aging, transcription, protein interaction

## Abstract

**Simple Summary:**

The tumor suppressor p53 is frequently mutated across numerous cancer types and has an essential role in the response to DNA-damaging agents, such as irradiation. DNA damage can induce different forms of the p53 protein, and the different forms of the p53 protein can elicit distinct types of cell death. Specifically, the activation of full-length p53 typically results in cellular apoptosis, while the induction of the alternatively spliced isoform, the beta isoform of p53 (p53β), results in cellular senescence. In this report, we review the known roles of p53β and introduce novel insights into p53β function.

**Abstract:**

Cellular responses to DNA damage and other stresses are important determinants of mutagenesis and impact the development of a wide range of human diseases. TP53 is highly mutated in human cancers and plays an essential role in stress responses and cell fate determination. A central dogma of p53 induction after DNA damage has been that the induction results from a transient increase in the half-life of the p53 protein. Our laboratory recently demonstrated that this long-standing paradigm is an incomplete picture of p53 regulation by uncovering a critical role for protein translational regulation in p53 induction after DNA damage. These investigations led to the discovery of a DNA-damage-induced alternative splicing (AS) pathway that affects p53 and other gene products. The damage-induced AS of p53 pre-mRNA generates the beta isoform of p53 (p53β) RNA and protein, which is specifically required for the induction of cellular senescence markers after ionizing irradiation (IR). In an attempt to elucidate the mechanisms behind the differential regulation and apparent functional divergence between full-length (FL) p53 and the p53β isoform (apoptosis versus senescence, respectively), we identified the differential transcriptome and protein interactome between these two proteins that may result from the unique 10-amino-acid tail in p53β protein.

## 1. Identification of Ribosomal Protein L26 (RPL26) as a Critical Mediator of p53 Translation after DNA Damage

Cellular responses to DNA damage and other stresses are important determinants of cell survival and ultimately impact the development of a wide range of human diseases, including cancer where TP53 remains the most frequently mutated gene across numerous cancer types. Specifically, the ability to properly modulate the response to stress impacts tumor development, tumor responses to therapy, the toxicities of cancer treatments, the development of cardiovascular disease, outcome (extent of organ damage) following a heart attack or stroke, and the rate of progression of certain neurodegenerative disorders. When cells properly respond to stressors, like irradiation (IR)-induced DNA damage, levels of p53 protein increase, thereby modulating cell cycle progression, cell death, and cellular senescence. Increases in p53 protein levels after DNA damage have largely been attributed to increases in the half-life of the p53 protein, primarily via the modulation of proteasome-mediated degradation of the p53 protein by the E3-ubiquitin ligase MDM2. However, we have demonstrated that increased translation of p53 mRNA is also a requisite step for optimal p53 induction following DNA damage [1,2,3]. 

Exploration of the mechanisms involved in the translational regulation of p53 after IR identified the ribosomal protein L26 (RPL26) and the nucleolar protein, nucleolin, as positive and negative regulators, respectively, of this process [3]. Moreover, the ubiquitin ligase MDM2, a known modulator of p53 protein degradation [4,5], regulates both RPL26 protein degradation and its binding to p53 mRNA [2]. Further, we found that p53 mRNA forms a double-stranded RNA structure created by base-pairing of complementary sequences of the 5′- and 3′-UTRs and that this structure is required for the binding of RPL26 and the regulation of p53 translation after stress [1,6]. In all, these studies demonstrated the critical extra-ribosomal role of RPL26 in the stress-regulated control of p53 translation.

## 2. Discovery of a DNA-Damage-Induced Alternative Splicing Pathway that Links the Beta Isoform of p53 (p53β) to Cellular Senescence

After the discovery that RPL26 was involved in increasing p53 translation after DNA damage, it seemed unlikely that p53 would be the only cellular gene product regulated after stress by such an elegant pathway. In order to investigate whether gene products other than p53 might be upregulated by translation in a similar fashion after DNA damage, we performed gene array analyses to identify RNA molecules that interact with the RPL26 protein following IR. One of these RNA products turned out to be the β (beta) isoform of p53 (p53β), an alternatively spliced form of p53 RNA. More than 10 different alternatively spliced forms of human p53 have been described, but we have a very limited understanding of the regulation and function of these isoforms [7]. Full-length p53 (FLp53) and p53β share their first 331 amino acids, including the DNA binding domain, but differ in that the C-terminal 62 amino acids of FLp53, which contains the oligomerization domain, are eliminated by alternative splicing in the p53β isoform and a novel 10-amino-acid segment is added to the protein (reviewed in [7]). As we conducted studies of IR-induced translational regulation of p53, we demonstrated an IR-induced increase in levels of the β isoform of p53 RNA and protein, suggesting a potential role for this p53 isoform in cellular responses to DNA damage [8].

**Figure 1:**
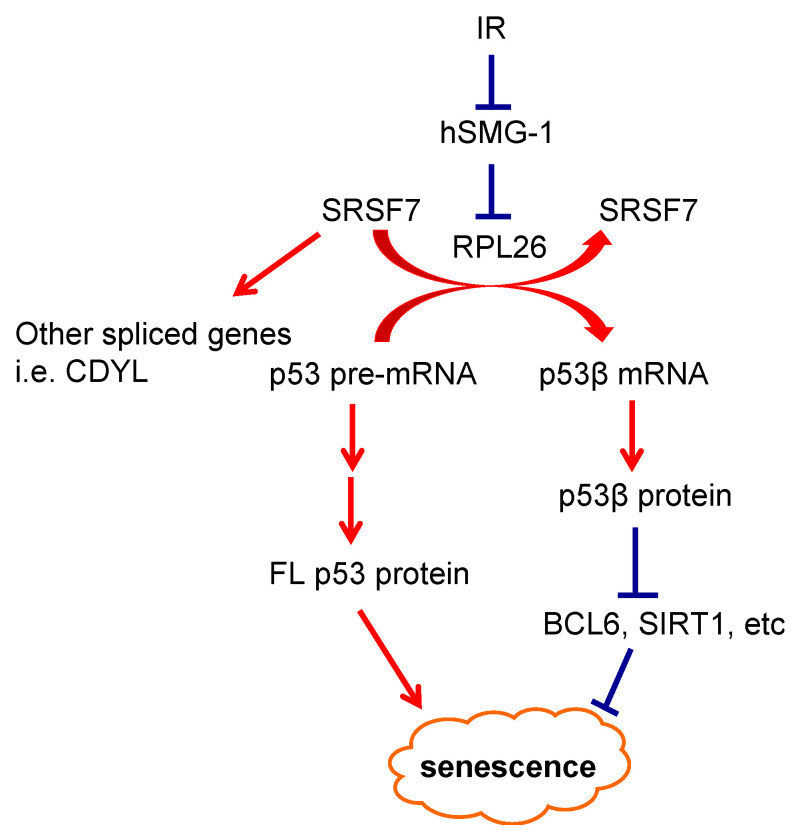
Schema of the new arm of the DNA damage signaling pathway (DDR). Irradiation (IR) suppresses hSMG1 kinase activity, inactive hSMG1 releases from p53 pre-mRNA and allows ribosomal protein L26 (RPL26)–SRSF7 to bind, resulting in splicing to generate p53β. p53β is indispensable for induction of senescence markers after IR, perhaps through transcriptional repression. (Adapted from Chen et al., Cancer Discovery, 2017 [8]).

Since ataxia-telangiectasia, mutated (ATM) protein kinase is required for optimal p53 induction after IR [9], we asked whether ATM was involved in this IR-induced splicing event. Using an inhibitor of ATM kinase activity that had been characterized by our laboratory [10,11,12], we found that exposure of cells to this kinase inhibitor induced, rather than inhibited, the generation of p53β. This observation led us to demonstrate that the induction of p53β after IR arose from the suppression of kinase activity of the ATM-related phosphatidylinositol 3-kinase-related kinase (PIKK) family member hSMG1. In addition, consistent with the observation that the binding of RPL26 to p53β RNA increases after IR, we found that RPL26 was also required for IR-induced alternative splicing of p53 pre-mRNA to p53β RNA. As RPL26 binds to p53 pre-mRNA after IR, it recruits the RNA splicing factor SRSF7, which is then able to induce the alternative splicing of p53 pre-mRNA to produce p53β mRNA. This series of protein–RNA binding changes is initiated by the IR-induced suppression of hSMG1 kinase activity and the selective release of hSMG1 from the exon/intron 9 region of p53 pre-mRNA. The release of hSMG1 from this site in p53 pre-mRNA then permits the binding of RPL26/SRSF7 to this same region of the RNA and leads to the alternative splicing that generates p53β mRNA. The induction of p53β after IR appeared to selectively regulate IR-induced cellular senescence (see below), establishing a specific physiologic signaling pathway and role for one of the p53 isoforms [8]. This represented a novel arm of the DNA damage signaling pathway (DDR) and was consistent with reported links of alternative splicing to both cancer [13] and aging [14]. A schema summarizing the steps involved in the IR-induced alternative splicing of p53 RNA to generate p53β RNA is illustrated in Figure 1.

## 3. Post-Transcriptional Regulation of p53β

In addition to the studies described above, wherein hSMG1 was identified as a negative regulator of p53β alternative splicing, RPL26 and SRSF7 were identified as positive regulators of p53β splicing in the DNA-damage-induced alternative splicing pathway. Other studies have associated additional RNA binding proteins in basal regulation of p53β expression. The splicing factor SRSF3 was shown to inhibit alternative splicing of the p53β-unique exon, identifying another negative regulator of p53β expression [15]. Moreover, the splicing factor SRSF1 was also identified as an additional negative regulator of p53β expression, as p53β levels increased upon SRSF1 inhibition or knockdown [16,17]. Finally, while alternative splicing is responsible for the expression of p53β, the act of incorporating the p53β-unique exon introduces a premature termination codon (PTC), which is typically recognized by nonsense-mediated RNA decay (NMD) machinery [18]. Consequently, the NMD factors UPF1 and SMG7 have been shown to increase p53β expression [17], which indicates that p53β could be a target for NMD. The balance between alternative splicing and NMD of p53β remains to be elucidated, especially in the presence of cellular stressors. Importantly, many of these studies were performed in the absence of insult, thus it remains unknown how these factors cooperate with the DNA-damage-induced alternative splicing pathway.

## 4. DNA Damage Induction of p53β Links to Cellular Senescence but not Apoptosis

**Figure 2:**
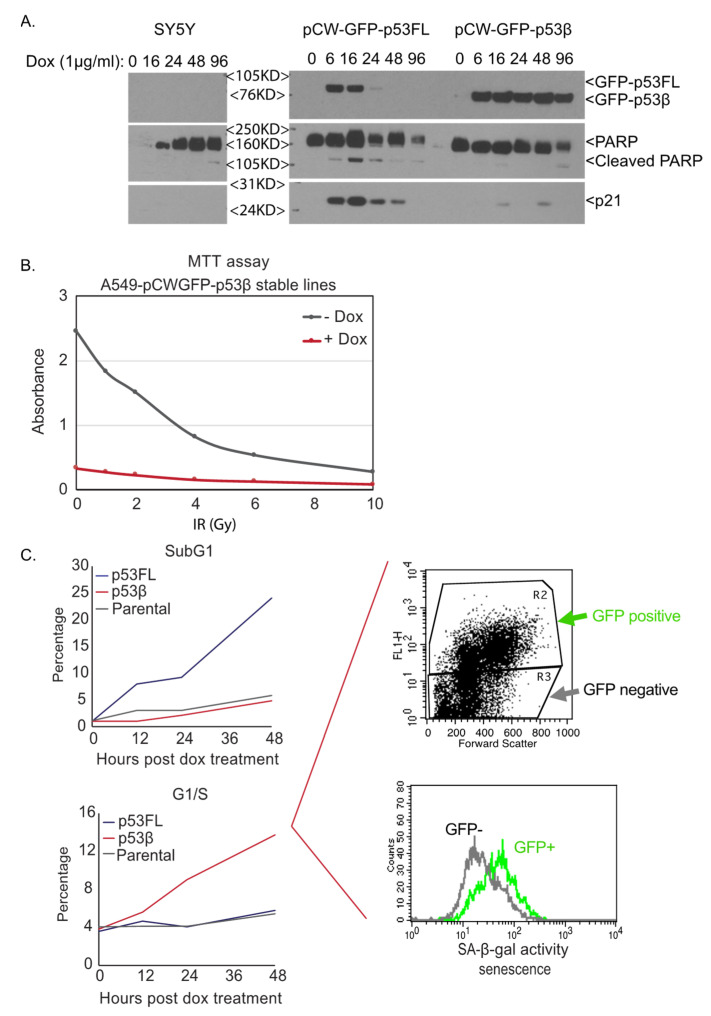
Modulation of growth arrest vs. apoptosis in p53-inducible cell lines. (**A**). GFP-tagged full-length p53 (FLp53) and p53β are induced in SH-SY5Y cells post doxycycline treatment. p21 induction and PARP cleavage are only observed in FLp53-overexpressing cells. Antibodies for WB in this figure: GFP (Abcam), PARP (Cell signaling), p21 (BD). (**B**). Synergistic effect of p53β overexpression and IR. GFP-p53β is induced to overexpress in the A549-GFP-p53β-inducible line by Doxycycline treatment for 24 h before subsequent IR at different doses. A549-GFP-p53β without GFP-p53β induction is used as a control. Cell proliferation is assessed by MTT assay 10 days post-IR [22]. (**C**). Differential cellular effects in FLp53 and p53β-induced cells. As assessed by PI staining for cell cycle analysis, FLp53-induced SY5Y cells undergo apoptosis as indicated by the increasing subG1 population (top left panel; blue line) while p53β-overexpressing cells (GFP-positive population, top right panel) are arrested at G1 (bottom left panel; increased G1/S ratio, red line) and stained positive for SA-β-galactosidase activity (bottom right), a classical cellular senescence marker in the flow beta-gal assay described in Chen et al. Cancer Discovery [8].

The DNA damage induction of the FLp53 protein results in either the arrest of cells in the G1 phase of the cell cycle [19] or the induction of apoptotic cell death [20], depending on cell type and other factors. In the limited studies on p53 isoforms, p53β was observed to increase in senescent cells, and induction was shown to decrease proliferation and increase cellular senescence [8,21]. Thus, in order to further explore the biological outcomes related specifically to p53β expression, FLp53 or p53β was transiently or inducibly overexpressed (Figure 2A). Doxycycline-induced expression of p53β decreased cell growth compared with the induced expression of FLp53 in the absence of insult as well as in an IR dose-dependent manner (Figure 2B). In the absence of insult, p53β expression caused a cellular senescent phenotype as indicated by G0/G1-phase growth arrest, SA-β-galactosidase (β-gal) positivity, senescence-associated secretory phenotype (SASP) induction, and p16INK4A induction, but there was no evidence of cell death (Figure 2C). In contrast, induction of FLp53 induced signs of both apoptotic cell death and senescence (Figure 2A,C). Since overexpression phenotypes can be misleading, we also generated cells in which p53β was specifically deleted by CRISPR-Cas9 mutagenesis; while these cells retained FLp53 expression and function, they were unable to produce p53β RNA and protein. Using these p53β-knockout cells, or by modulation of other pathway regulators (e.g., SMG1, SRSF7), we demonstrated that p53β was required for IR-induced cellular senescence in a manner dependent on SMG1, SRSF7, and p53β [8] (Figure 1 and Figure 2). 

## 5. p53β Expression is Associated with Better Clinical Outcomes

**Figure 3:**
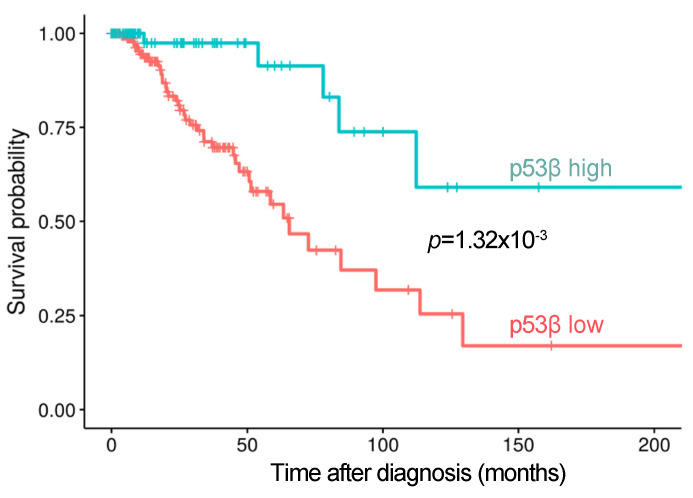
High levels of p53β are associated with prolonged overall survival in patients with advanced stage (III/IV) breast cancer (TCGA breast cancer (BRCA) data [24,25]). *N* = 277; unadjusted *p*-value = 0.00132. The “expression low” cohort represents the following combination of samples: HER2 negative (39.6%, *N* = 80), HER2 positive (17.3%, *N* = 35), NA (36.6%; *N* = 74; including HER2 equivocal), and triple-negative breast cancer (6.4%, *N* = 13). The “expression high” cohort represents the following combination of samples: HER2 negative (41.3%, *N* = 31), HER2 positive (16.0%, *N* = 12), NA (32.0%; *N* = 24; including HER2 unequivocal), and triple-negative breast cancer (10.7%, *N* = 8). The cohort was 32.5% (*N* = 90) TP53 mutant and 67.5% (*N* = 187) TP53 wild type (WT). HER2-negative status was determined as; (1) ER−, PR+; (2) ER+, PR−; (3) ER+, PR+; or (4) ER+, PR indeterminate. Methods for data analysis can be found in supplementary material.

In addition to our discovery that the induction of p53β is specifically required for IR-induction of cellular senescence markers, sporadic reports also suggest that the expression of p53 isoforms is induced in cancer cell lines treated with DNA damage reagents and related to cancer clinical features and outcome [23]. In order to explore whether expression or induction of p53β could serve as a predictive biomarker of outcome in cancer patients treated with radiation or chemotherapy, we interrogated the TCGA database and corresponding RNA-Sequence data of the patients and found evidence suggesting that in stage III/IV breast cancer patients, who are commonly treated with chemotherapy and/or radiation therapy, high levels of p53β RNA expression were associated with prolonged overall survival (Figure 3). These observations support the potential clinical importance of p53β and further investigation into this alternative splicing pathway.

## 6. Differential p53β Target Genes

At least some of the cellular effects of p53β appear to require its transcriptional regulatory activity since mutation of the DNA binding domain of p53β attenuated its ability to induce cellular senescence markers [8]. Sporadic reports in the literature have suggested that p53β regulates known p53 target genes in a promoter-dependent manner [16,26]. Our data demonstrate that the overexpression of p53β only impacts a subset of p53 target genes [8], which suggests that the transcriptional activities of p53β protein exhibit both similarities and differences from FLp53 protein. On one hand, as a transcription factor, p53β is able to transcriptionally activate certain p53 target genes, either in an FLp53-independent (such as TP53INP1) or an FLp53-dependent manner (such as GDF15), as determined through microarray analysis [8]. On the other hand, p53β may be able to repress the expression of other gene products, such as the ‘anti-aging’ genes BCL6 and SIRT, perhaps contributing to the role of p53β in IR-induced cellular senescence [8]. It is conceivable that global mapping and comparison of p53β and FLp53 genomic DNA binding sites coupled with RNA sequencing will be an important step in deciphering differential transcriptional activities of these two proteins. Moreover, the definition of the p53β cistrome will also lend insight into direct targets of p53β as well as potential transcriptional co-regulators of p53β activity.

## 7. Differential p53β Protein Interactors

**Figure 4:**
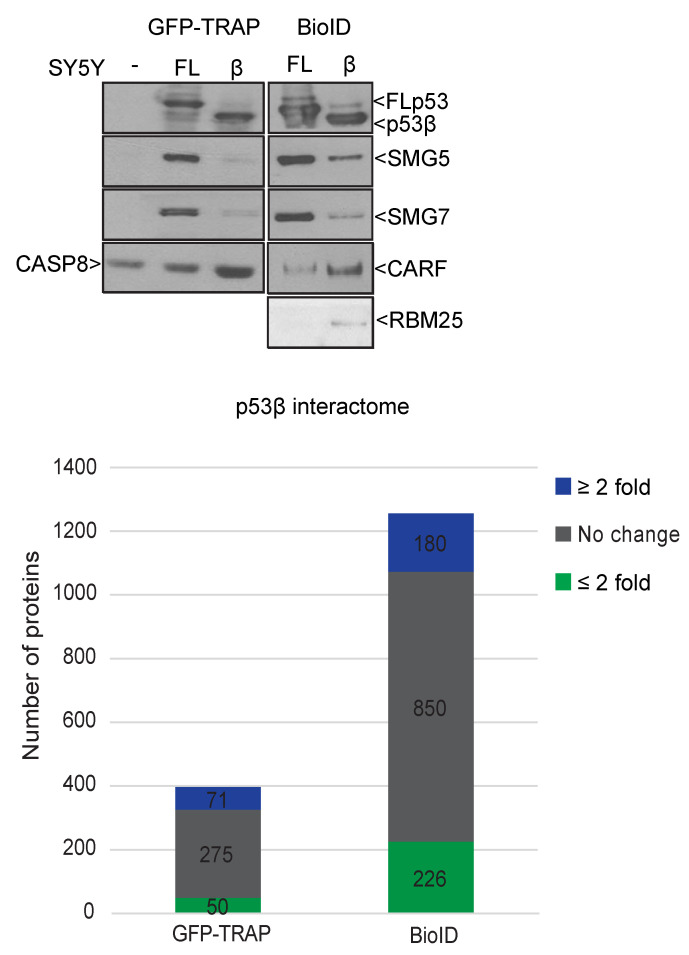
Probing the p53β interactome by the GFP-TRAP system and p53 BioID. Left: Validation of hits from the GFP-TRAP system performed in SY5Y-inducible cell lines (left panels) and from p53 BioID system in MCF7 cells (right panels). Right: Bar graph shows a summary of the number of proteins identified in the GFP-TRAP and BioID systems. Blue: the number of proteins that bind ≥2 fold more to p53β than to FLp53; green: the number of proteins that bind ≥2 fold less to p53β than to FLp53; and grey: the number of proteins in between.

The shortened/altered C-terminal tail of p53β is the only difference between p53β and FLp53 in terms of the linear amino acid sequence. The C-terminal domain of FLp53 protein contains several sites for post-translational modifications, especially acetylation and ubiquitination, as well as domains for protein–protein interactions and oligomerization. It will be intriguing to explore how the replacement of the C-terminal tail of FLp53 by a short, new 10-amino-acid sequence in p53β will change the protein interactome of p53β and to determine which changes in this interactome contribute to the distinct cellular effects of p53β compared to FLp53, specifically the senescent phenotype versus programmed cell death. To begin to address these questions, we used two independent and complementary systems of p53 expression: GFP-TRAP and BioID. For the former, we generated GFP-FLp53-inducible and GFP-p53β-inducible SY5Y cell lines. After doxycycline-induction, GFP-FLp53 and GFP-p53β proteins were pulled down by GFP-TRAP beads and bound proteins were identified by quantitative mass spectrometry (MS). This system permits the determination of differences in strong interactors of the FLp53 protein versus the p53β protein. Initial MS screening after GFP-p53FL or GFP-p53β pulldowns identified proteins such as SMG5, SMG7, MDM2, and MDM4 as interactors with GFP-p53FL, but not GFP-p53β (Figure 4, left). These proteins all require the p53 oligomerization domain (lacking in p53β protein) for interaction, so they serve as reassuring positive controls for the system’s ability to successfully identify differential binding partners. Interestingly, about 71 proteins had ≥2 fold more binding to p53β than to FLp53 and 50 proteins showed ≥2 fold less binding to p53β than to FLp53. Thus, differential cofactor binding occurs between p53β and FLp53.

As a complementary approach, we adapted the BioID system [27] by fusing FLp53 or p53β coding sequences with BirA, a bacterial promiscuous biotin ligase. BirA–FLp53 and BirA–p53β fusion proteins expressed in cells biotinylate proximate and interacting proteins in the presence of ATP and biotin, allowing for identification and quantification by MS. While the TRAP system or other co-IP approaches can be effective at identifying strongly interactive proteins, the BioID system serves as a complementary approach in that it can identify more transiently bound cofactors. Preliminary identification of the FLp53 and p53β interactomes identified 180 proteins that demonstrate greater binding to p53β than to FLp53. Confirming results from the GFP-TRAP system, the BioID system also showed that SMG5 and SMG7 preferentially bind to FLp53 relative to p53β (Figure 4). It is noted that the NMD regulator, SMG7, was recently shown to regulate p53β levels via modulation of nonsense-mediated RNA decay (27). Among the 180 potential interactors, CARF and RBM25 were validated as preferential p53β interactors. Thus, replacing the oligomerization domain of FLp53 with the short 10-amino-acid tail in p53β does alter the binding spectrum of the protein and may impart “new” functional properties (gain-of-function) to p53β. Interestingly, the pathway analysis to date on these differential binding partners suggests that p53β protein binds more than FLp53 to proteins functioning in RNA processing, especially spliceosome components.

## 8. Conclusions: Future Directions and Unanswered Questions

The functional differences we and others have observed between FLp53 protein and its splice variant, p53β, could conceivably result from at least two different general mechanisms: (1) differential transcriptional target gene expression, either resulting from: (a) differential binding of FLp53 vs. p53β to cis-regulatory sequences for regulating gene expression, (b) competition between FLp53 and p53β proteins for the same DNA regulatory sequences (with tetrameric FLp53 being the more potent transcriptional activator as p53β as a competitor could reduce transactivation by FLp53 by binding to the same sites), and/or (c) differential binding of FLp53 vs. p53β to transcriptional co-factors, regulating strength of transactivation or even whether transcriptional activation vs. transcriptional repression is induced; or (2) differential functions of FLp53 vs. p53β in regulating non-transcriptional events, such as differential interactions with cell-cycle or apoptosis-regulating proteins. Whether the functional differences between FLp53 and p53β are a result of differential DNA binding or differential protein–protein interactions, such functional differences could result from differential post-translational modifications of FLp53 vs. p53β, the lack of the C-terminal tail in p53β, or from distinct activities gained from the presence of the unique C terminal tail or its post-translational modifications. Data from our lab and others have shown that differential transcriptional target gene expression does occur [8,26]. Global mapping of FLp53 and p53β binding sites compared to differential transcriptome regulation will begin to elucidate the differential regulation put forth by either protein. Finally, we presented evidence that differential cofactor binding does occur. In all, p53β promotes pro-senescent phenotypes, thus promoting differential molecular and biological outcomes compared with FL-p53.

## 9. Patents

Not applicable.

## 10. Materials and Methods:

### 10.1. Cell Culture, Stable Cell Lines, and Transfection

Parental A549 and MCF-7 cells were maintained in DMEM plus 10% FBS and 1% antibiotics. Parental SH-SY5Y cells were maintained in RPMI supplemented with 15% FBS plus 1% antibiotics. SH-SY5Y and A549-inducible cell lines to express GFP-tagged full-length human p53 (GFP-p53FL) or p53β were generated by infection of lentiviruses and selected with 2 μg/mL of puromycin. After selection, stable lines were maintained in appropriate media supplemented with 1 μg/mL of puromycin. MCF-7 cells were transfected with pCMV-BirA-p53FL or pCMV-BirA-p53β for BioID experiments by Lipofectamine 2000 (Thermofisher Waltham, MA, USA). MCF-7 (date of purchase, 18 April 2006), SY5Y (date of purchase, 12 February 1999), and A549 (date of purchase, 7 February 2006) were purchased from the ATCC between the years 1999 and 2009 and authenticated on 29 August 2016, using certified human cell line authentication (CLA) analysis provided by the Duke University DNA analysis facility.

### 10.2. Plasmids and Chemicals

Plasmids used in this study for viral packaging and generation of stable inducible lines include pCW-GFP-p53FL, pCW-GFP-p53β, pCMV-BirA-p53FL, and pCMV-BirA-p53β. Doxycycline and Biotin were purchased from Sigma (St. Louis, MO, USA).

### 10.3. Immunoblot and Immunoprecipitation Using GFP-TRAP^®^ and BioID

Cell lysates were prepared by a freeze–thaw, followed by incubation in RIPA buffer for 30 min on ice, and the supernatants were analyzed by immunoblot analysis or immunoprecipitation. For immunoblot analysis, 20 to 50 μg protein samples were denatured in an equal volume of SDS sample buffer (BioRad, Hercules, CA, USA), separated by 4% to 12% SDS-PAGE, and transferred to nitrocellulose membrane. The blots were probed with a primary antibody (1:1000 or at concentrations recommended by the manufacturers) against p53 (DO-1; Santa Cruz Biotechnology, Dallas, TX, USA), PARP (Cell Signaling Technology, Danvers, MA, USA), p21 (BD Pharmingen San Diego, CA, USA), SMG5, SMG7, CARF and RBM25 (Bethyl Laboratories, Montgomery, TX, USA), and CASP8 (Abcam Cambridge, UK). Primary antibody binding was detected by incubating with horseradish peroxidase (HRP)-conjugated anti-rabbit and anti-mouse secondary antibody (1:10,000 dilution; Rockland Inc., Gilbertsville, PA, USA) followed by enhanced chemiluminescent (ECL) visualization (Amersham Biosciences Little Chalfont, UK).

At 24 h post doxycycline induction, GFP-tagged proteins were immunoprecipitated from SY5Y-inducible cell line lysates by GFP-TRAP^®^ agarose beads (Chromotek Munich, Germany) according to the manufacturer’s recommendations. The eluted GFP-tagged proteins and their associated proteins were subjected to MS performed by the Duke Proteomics and Metabolomics Shared Resource. On-resin digestion of the GFP immunocomplex and quantitative proteomic analysis of these samples was performed followed by quantitative MS.

At 6 h post transient transfection of MCF-7 with pCMV-BirA constructs, Biotin was added to the cell culture for overnight labeling. At 24 h post-transfection, transfected cells were harvested for cell lysate preparation and immunoprecipitated by streptavidin beads (Amersham Little Chalfont, UK). The enriched biotinylated proteins were subjected to mass spectrometry performed by the Duke Proteomics and Metabolomics Shared Resource.

As indicated above, Streptavidin and GFP-Trap eluents were analyzed by the Duke Proteomics and Metabolomics Shared Resource. Briefly, samples were reduced in loading buffer and separated by SDS-PAGE for 3–4 min followed by Coomassie staining and in-gel digestion with trypsin. Digests were analyzed by LC-MS/MS using a Waters nanoACQUITY UPLC interfaced to a Thermo Fusion Lumos MS. The LC separation 90 min gradient of 5–30% MeCN in a trapping configuration with a 75 um × 25 cm analytical column (Waters HSS T3). MS/MS analysis used orbitrap MS1 and ion trap MS2 (OT-IT). Raw files were converted to mgf using Proteome Discoverer and searched against a Swiss-Prot human database appended with the p53β sequence and an equal number of reverse decoys, with 5 ppm precursor and 0.8 Da product ion tolerances, trypsin specificity with up to 2 missed cleavages, fixed carbamidomethyl modification on Cys and variable deamidation (NQ), oxidation (M), peptide N-terminal Gln → pyroGlu, and protein N-terminal acetylation. Data were annotated at a 1% peptide and protein false discovery rate (FDR) using Scaffold. The raw MS files and Scaffold files were deposited to ProteomeXchange with identifier PXD023327 via the MassIVE repository.

### 10.4. Flow SA-β-Galactosidase Assay

The flow SA-β-Galactosidase assay was adapted from [8]. In brief, SY5Y-inducible cell lines were treated with 1 µg/mL doxycycline for the indicated time before being subjected to PI staining or flow SA-β-Gal assay. For the flow SA-β-Gal assay, sub-confluent cells were first treated with 100 nmol/L bafilomycin A1 (Sigma) for 1 h in fresh DMEM plus 10% FBS and 1% antibiotics medium at 37 °C, 5% CO_2_. A final concentration of 33 μmol/L DDAOG (Setareh Biotech Eugene, OR, USA) was added to the medium for an extra 2 h of incubation. The cell monolayer was then washed twice with room-temperature PBS and harvested by trypsinization followed by centrifugation at 200× *g* for 10′ at 4 °C. Cells were resuspended in ice-cold PBS at a concentration of 1 × 10^6^ cells/mL and run immediately on BDCalibar. Data were collected and analyzed using CellQuest following the instructions of the manufacturer’s manual.

## Data Availability

The raw MS files and Scaffold files (Figure 4) were deposited to ProteomeXchange with identifier PXD023327 via the MassIVE repository.

## Supplementary Materials

The following are available online at https://www.mdpi.com/2072-6694/13/2/251/s1: Supplementary Data: Supplementary Materials and Methods and Supplementary References.

## References

[1] Chen J., Kastan M.B. (2010). 5′-3′-UTR interactions regulate p53 mRNA translation and provide a target for modulating p53 induction after DNA damage. Genes. Dev..

[2] Ofir-Rosenfeld Y., Boggs K., Michael D., Kastan M.B., Oren M. (2008). Mdm2 regulates p53 mRNA translation through inhibitory interactions with ribosomal protein L26. Mol. Cell.

[3] Takagi M., Absalon M.J., McLure K.G., Kastan M.B. (2005). Regulation of p53 translation and induction after DNA damage by ribosomal protein L26 and nucleolin. Cell.

[4] Honda R., Tanaka H., Yasuda H. (1997). Oncoprotein MDM2 is a ubiquitin ligase E3 for tumor suppressor p53. FEBS Lett..

[5] Ashcroft M., Vousden K.H. (1999). Regulation of p53 stability. Oncogene.

[6] Chen J., Guo K., Kastan M.B. (2012). Interactions of nucleolin and ribosomal protein L26 (RPL26) in translational control of human p53 mRNA. J. Biol. Chem..

[7] Joruiz S.M., Bourdon J.C. (2016). p53 Isoforms: Key Regulators of the Cell Fate Decision. Cold Spring Harb. Perspect. Med..

[8] Chen J., Crutchley J., Zhang D., Owzar K., Kastan M.B. (2017). Identification of a DNA Damage-Induced Alternative Splicing Pathway That Regulates p53 and Cellular Senescence Markers. Cancer Discov..

[9] Canman C.E., Kastan M.B. (1998). Small contribution of G1 checkpoint control manipulation to modulation of p53-mediated apoptosis. Oncogene.

[10] Min J., Guo K., Suryadevara P.K., Zhu F., Holbrook G., Chen Y., Feau C., Young B.M., Lemoff A., Connelly M.C. (2016). Optimization of a Novel Series of Ataxia-Telangiectasia Mutated Kinase Inhibitors as Potential Radiosensitizing Agents. J. Med. Chem..

[11] Guo K., Shelat A.A., Guy R.K., Kastan M.B. (2014). Development of a cell-based, high-throughput screening assay for ATM kinase inhibitors. J. Biomol. Screen..

[12] Rainey M.D., Charlton M.E., Stanton R.V., Kastan M.B. (2008). Transient inhibition of ATM kinase is sufficient to enhance cellular sensitivity to ionizing radiation. Cancer Res..

[13] David C.J., Manley J.L. (2010). Alternative pre-mRNA splicing regulation in cancer: Pathways and programs unhinged. Genes. Dev..

[14] Deschenes M., Chabot B. (2017). The emerging role of alternative splicing in senescence and aging. Aging Cell.

[15] Tang Y., Horikawa I., Ajiro M., Robles A.I., Fujita K., Mondal A.M., Stauffer J.K., Zheng Z.M., Harris C.C. (2013). Downregulation of splicing factor SRSF3 induces p53beta, an alternatively spliced isoform of p53 that promotes cellular senescence. Oncogene.

[16] Marcel V., Fernandes K., Terrier O., Lane D.P., Bourdon J.C. (2014). Modulation of p53beta and p53gamma expression by regulating the alternative splicing of TP53 gene modifies cellular response. Cell Death Differ..

[17] Cowen L.E., Luo H., Tang Y. (2019). Characterization of SMG7 14-3-3-like domain reveals phosphoserine binding-independent regulation of p53 and UPF1. Sci. Rep..

[18] Kurosaki T., Popp M.W., Maquat L.E. (2019). Quality and quantity control of gene expression by nonsense-mediated mRNA decay. Nat. Rev. Mol. Cell Biol..

[19] Kastan M.B., Onyekwere O., Sidransky D., Vogelstein B., Craig R.W. (1991). Participation of p53 protein in the cellular response to DNA damage. Cancer Res..

[20] Yonish-Rouach E., Resnitzky D., Lotem J., Sachs L., Kimchi A., Oren M. (1991). Wild-type p53 induces apoptosis of myeloid leukaemic cells that is inhibited by interleukin-6. Nature.

[21] Fujita K., Mondal A.M., Horikawa I., Nguyen G.H., Kumamoto K., Sohn J.J., Bowman E.D., Mathe E.A., Schetter A.J., Pine S.R. (2009). p53 isoforms Delta133p53 and p53beta are endogenous regulators of replicative cellular senescence. Nat. Cell Biol..

[22] Morgan D.M. (1998). Tetrazolium (MTT) assay for cellular viability and activity. Methods Mol. Biol..

[23] Avery-Kiejda K.A., Morten B., Wong-Brown M.W., Mathe A., Scott R.J. (2014). The relative mRNA expression of p53 isoforms in breast cancer is associated with clinical features and outcome. Carcinogenesis.

[24] The Cancer Genome Atlas Network (2012). Comprehensive molecular portraits of human breast tumours. Nature.

[25] Ciriello G., Gatza M.L., Beck A.H., Wilkerson M.D., Rhie S.K., Pastore A., Zhang H., McLellan M., Yau C., Kandoth C. (2015). Comprehensive Molecular Portraits of Invasive Lobular Breast Cancer. Cell..

[26] Bourdon J.C., Fernandes K., Murray-Zmijewski F., Liu G., Diot A., Xirodimas D.P., Saville M.K., Lane D.P. (2005). p53 isoforms can regulate p53 transcriptional activity. Genes Dev..

[27] Roux K.J., Kim D.I., Burke B. (2013). BioID: A screen for protein-protein interactions. Curr. Protoc Protein Sci..

