# Supplementary material for: DNA-Damage-Induced Alternative Splicing of p53
Source: Cancers (Basel). 2021 Jan 12;13(2):251. doi: 10.3390/cancers13020251 (PMC7827558; doi:10.3390/cancers13020251)
Supplement: Supplementary file 1 [file cancers-13-00251-s001.zip › Supplementary Data 1.pdf]

Supplementary Materials and Methods:

Supplemental Bioinformatics and Biostatistics Methods (Figure 3)

Raw RNA sequencing data in BAM format and clinical data for TCGA-BRCA [1,2] project were downloaded from Genomic Data Commons (GDC)[3] using R (v3.4.4)[4] and extension package TCGAbiolinks (v2.6.12)[5]. Aligned reads were assembled into transcripts and quantified using Stringtie (v1.3.0)[6]. The annotation file was obtained from the GDC portal (https://gdc.cancer.gov/about-data/data-harmonization-and-generation/gdc-reference-files). The read count matrices were normalized and transformed using edgeR (v3.20.9)[7] and limma (v3.34.9)[8]. For the isoform p53β, its normalized counts were dichotomized into high and low levels by splitting the values at the cutoff selected by conditional [9] method from the R package partykit (v1.2-0)[10], and a Cox model was used to regress overall survival outcome on p53β levels in the patients with stage III/IV breast tumors. The asymptotic P-value presented has not been adjusted for multiple testing or for measurement errors in the isoform quantification from the sequencing reads

Supplementary References:

1. Cancer Genome Atlas, N. Comprehensive molecular portraits of human breast tumours. Nature 2012, 490, 61-70, doi:10.1038/nature11412.
2. Ciriello, G.; Gatza, M.L.; Beck, A.H.; Wilkerson, M.D.; Rhie, S.K.; Pastore, A.; Zhang, H.; McLellan, M.; Yau, C.; Kandoth, C., et al. Comprehensive Molecular Portraits of Invasive Lobular Breast Cancer. Cell 2015, 163, 506-519, doi:10.1016/j.cell.2015.09.033.
3. Grossman, R.L.; Heath, A.P.; Ferretti, V.; Varmus, H.E.; Lowy, D.R.; Kibbe, W.A.; Staudt, L.M. Toward a shared vision for cancer genomic data. New England Journal of Medicine 2016, 375, 1109-1112.
4. R Core Team. R: A Language and Environment for Statistical Computing. Vienna, Austria, 2019.
5. Colaprico, A.; Silva, T.C.; Olsen, C.; Garofano, L.; Cava, C.; Garolini, D.; Sabedot, T.S.; Malta, T.M.; Pagnotta, S.M.; Castiglioni, I. TCGAbiolinks: an R/Bioconductor package for integrative analysis of TCGA data. Nucleic acids research 2015, 44, e71-e71.
6. Pertea, M.; Pertea, G.M.; Antonescu, C.M.; Chang, T.-C.; Mendell, J.T.; Salzberg, S.L. StringTie enables improved reconstruction of a transcriptome from RNA-seq reads. Nature biotechnology 2015, 33, 290.
7. Robinson, M.D.; McCarthy, D.J.; Smyth, G.K. edgeR: a Bioconductor package for differential expression analysis of digital gene expression data. Bioinformatics 2010, 26, 139-140.
8. Ritchie, M.E.; Phipson, B.; Wu, D.; Hu, Y.; Law, C.W.; Shi, W.; Smyth, G.K. limma powers differential expression analyses for RNA-sequencing and microarray studies. Nucleic acids research 2015, 43, e47-e47.
9. Hothorn, T.; Hornik, K.; Zeileis, A. Unbiased recursive partitioning: A conditional inference framework. Journal of Computational and Graphical statistics 2006, 15, 651-674.
10. Hothorn, T.; Zeileis, A. partykit: A modular toolkit for recursive partytioning in R. The Journal of Machine Learning Research 2015, 16, 3905-3909.
