# Supplementary material for: DNA-Damage-Induced Alternative Splicing of p53
Source: Cancers (Basel). 2021 Jan 12;13(2):251. doi: 10.3390/cancers13020251 (PMC7827558; doi:10.3390/cancers13020251)
Supplement: Supplementary file 1 [file cancers-13-00251-s001.zip › Supplementary Data 2.pdf]

Figure 2A

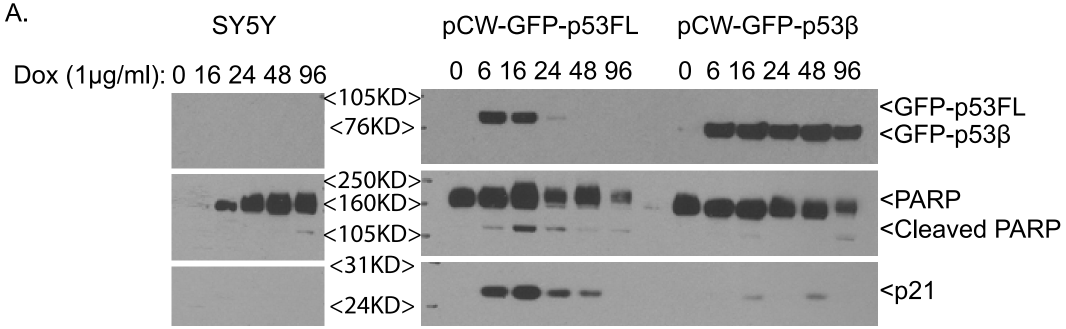
A.
SY5Y
pCW-GFP-p53FL
pCW-GFP-p53β
Dox (1µg/ml): 0 16 24 48 96
0 6 16 24 48 96
0 6 16 24 48 96
<105KD>
<76KD>
<250KD>
<160KD>
<105KD>
<31KD>
<24KD>
<GFP-p53FL
<GFP-p53β
<PARP
<Cleaved PARP
<p21

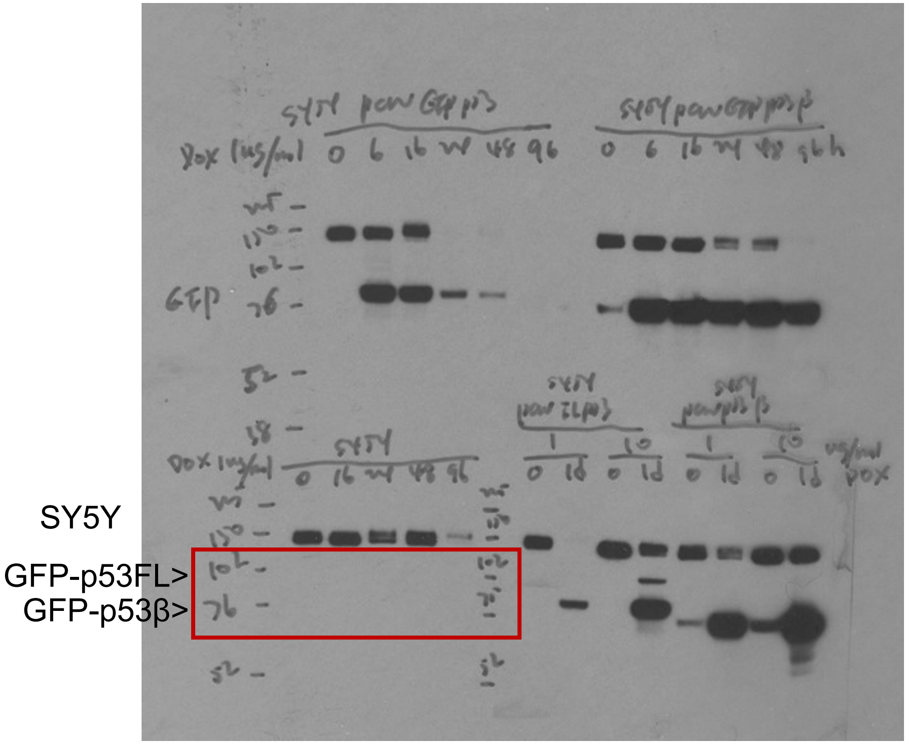
SY5Y
GFP-p53FL>
GFP-p53β>

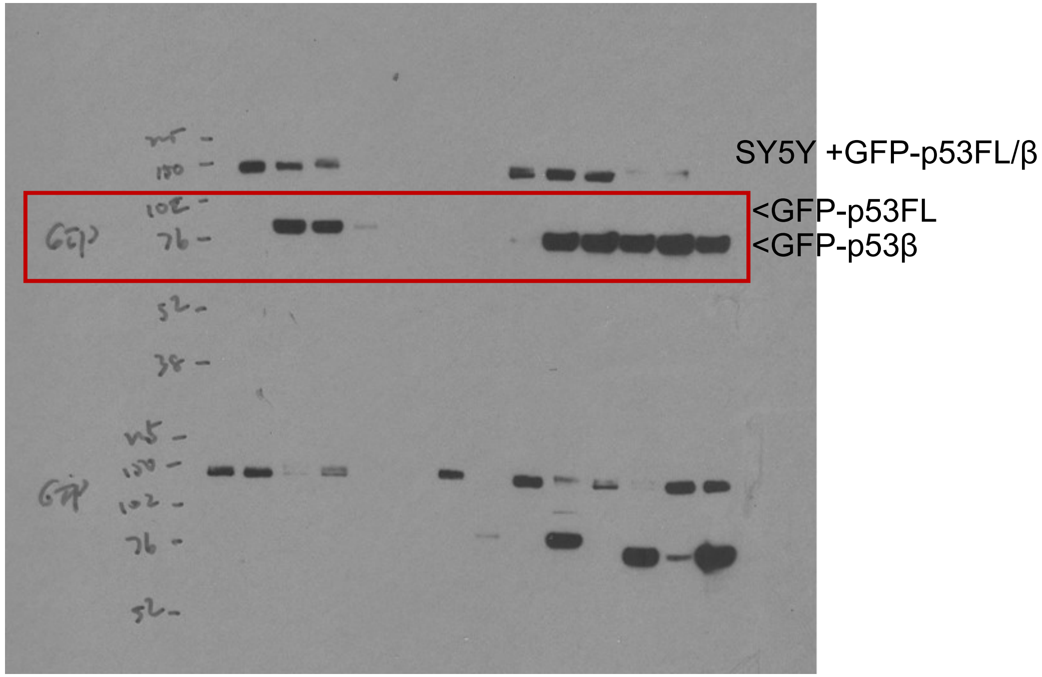
SY5Y +GFP-p53FL/β
<GFP-p53FL
<GFP-p53β

Figure 2A

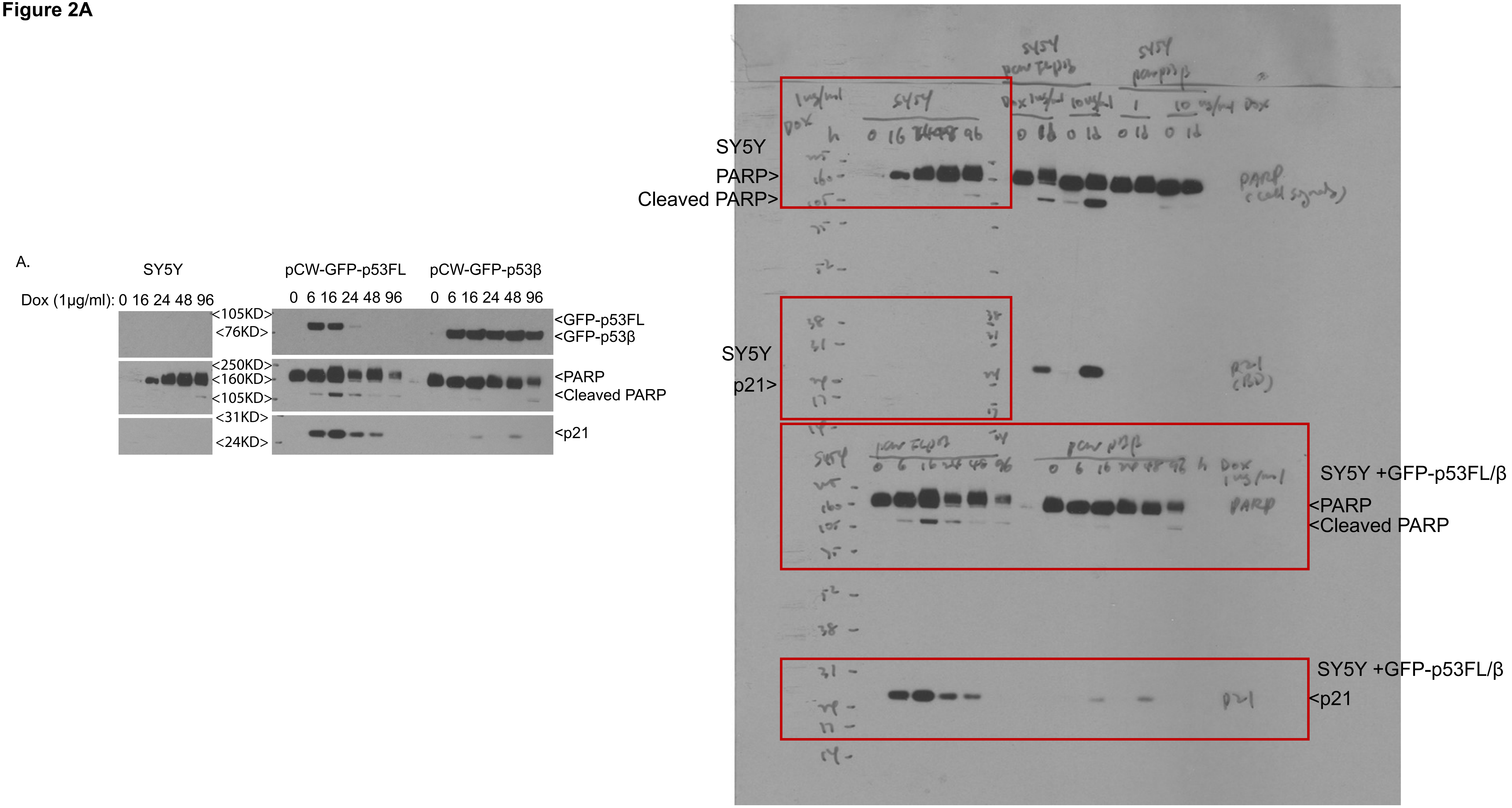
A.
SY5Y
pCW-GFP-p53FL
pCW-GFP-p53β
Dox (1μg/ml): 0 16 24 48 96
0 6 16 24 48 96
0 6 16 24 48 96
<105KD>
<76KD>
<250KD>
<160KD>
<105KD>
<31KD>
<24KD>
<GFP-p53FL
<GFP-p53β
<PARP
<Cleaved PARP
<p21
SY5Y
PARP>
Cleaved PARP>
SY5Y
p21>
SY5Y +GFP-p53FL/β
<PARP
<Cleaved PARP
SY5Y +GFP-p53FL/β
<p21

Figure 4A

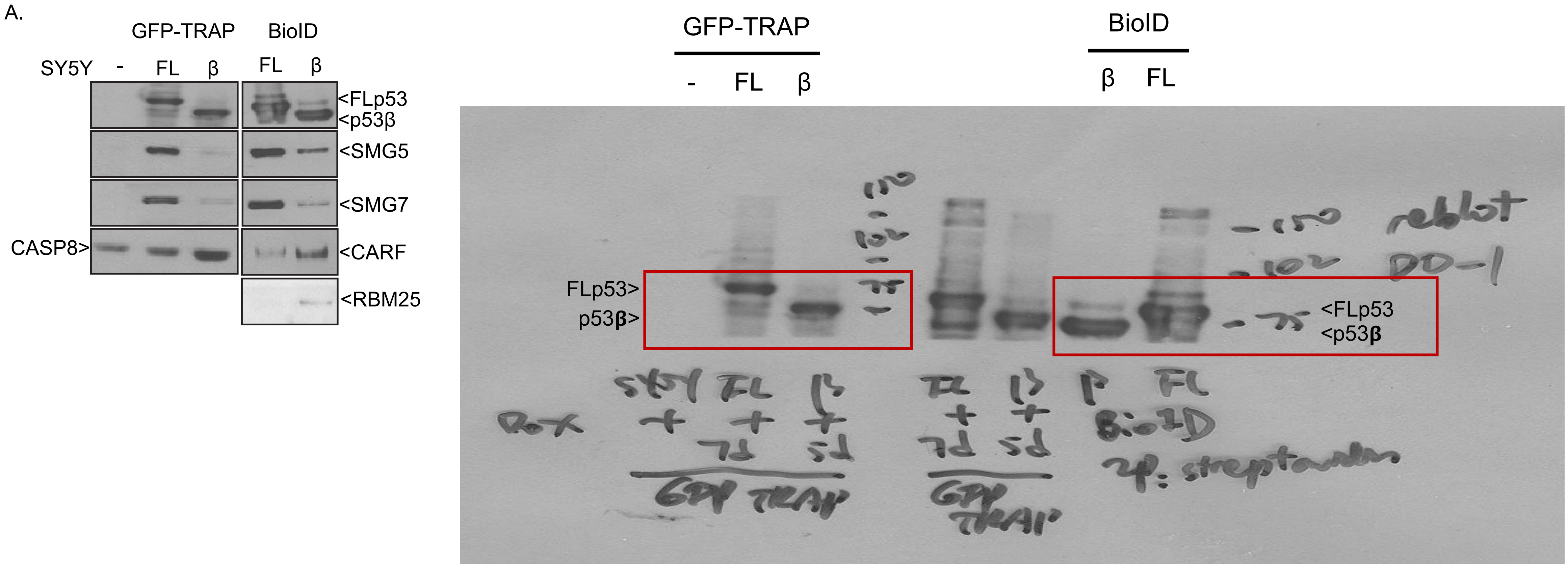

A.
GFP-TRAP
BioID
SY5Y
-
FL
β
FL
β
<FLp53
<p53β
<SMG5
<SMG7
CASP8>
<CARF
<RBM25
GFP-TRAP
-
FL
β
BioID
β
FL
FLp53>
p53β>
<FLp53
<p53β

Figure 4A

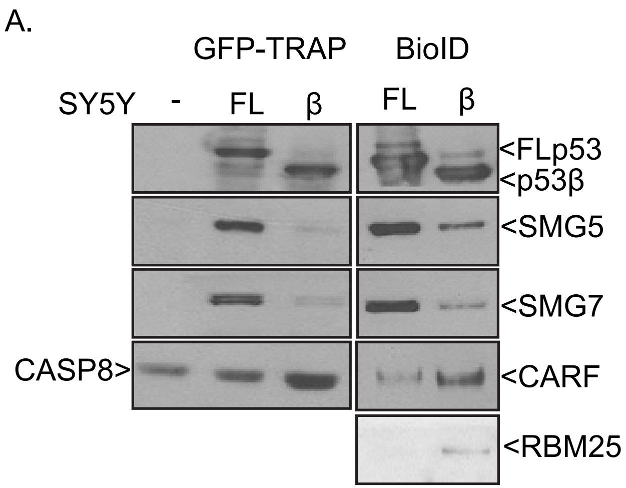

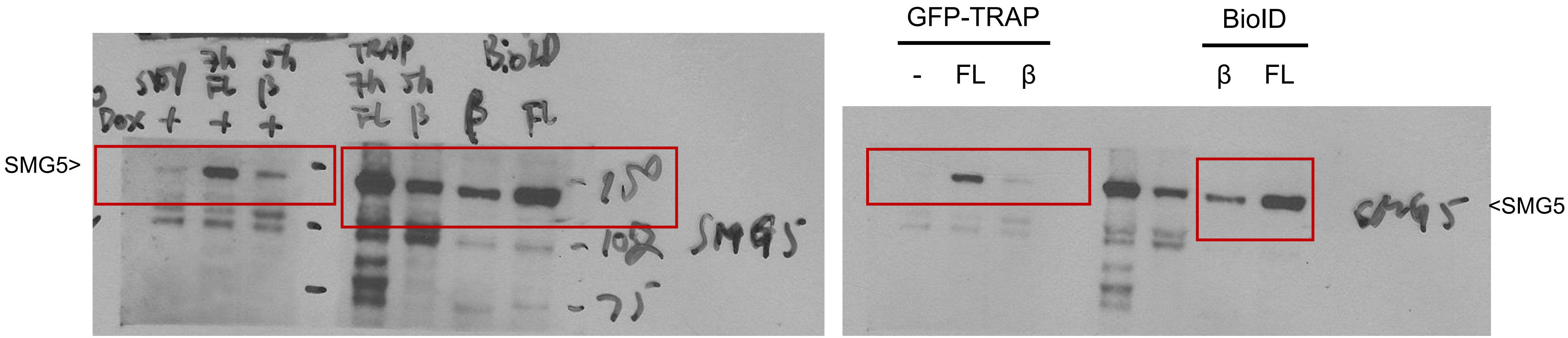

Longer exposure with markers

Figure 4A

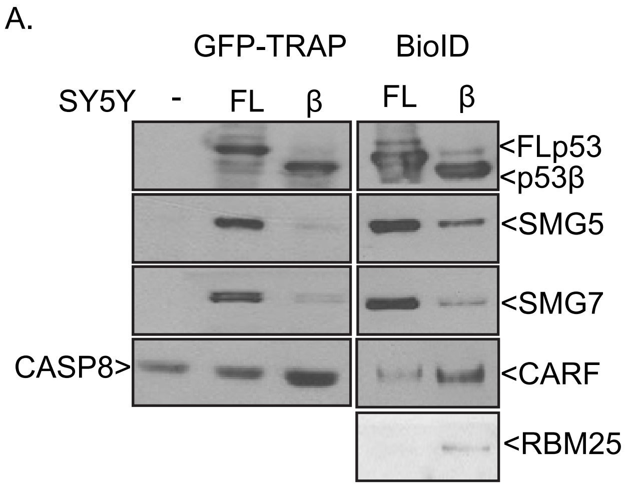
A.
GFP-TRAP
BioID
SY5Y
-
FL
β
FL
β
<FLp53
<p53β
<SMG5
<SMG7
CASP8>
<CARF
<RBM25

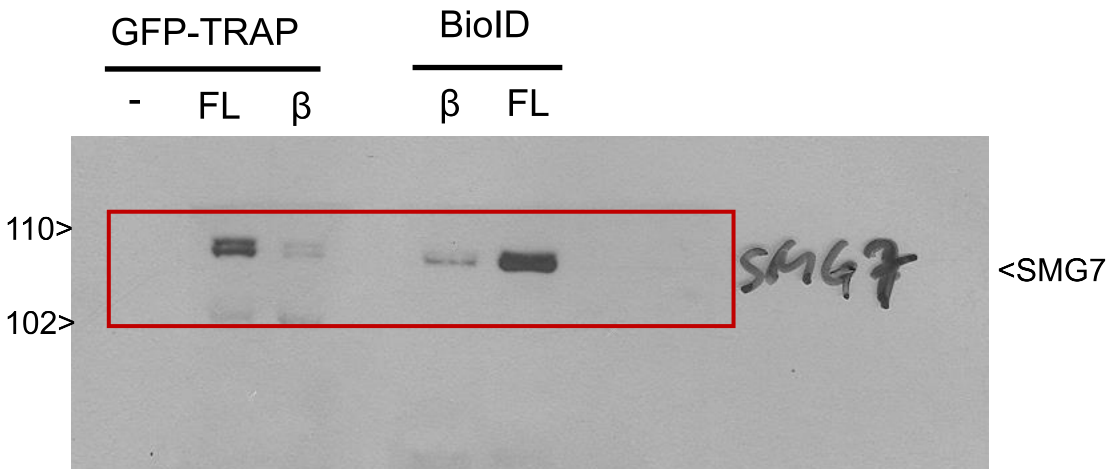
GFP-TRAP
BioID
-
FL
β
β
FL
110>
102>
SMG7
<SMG7

Figure 4A

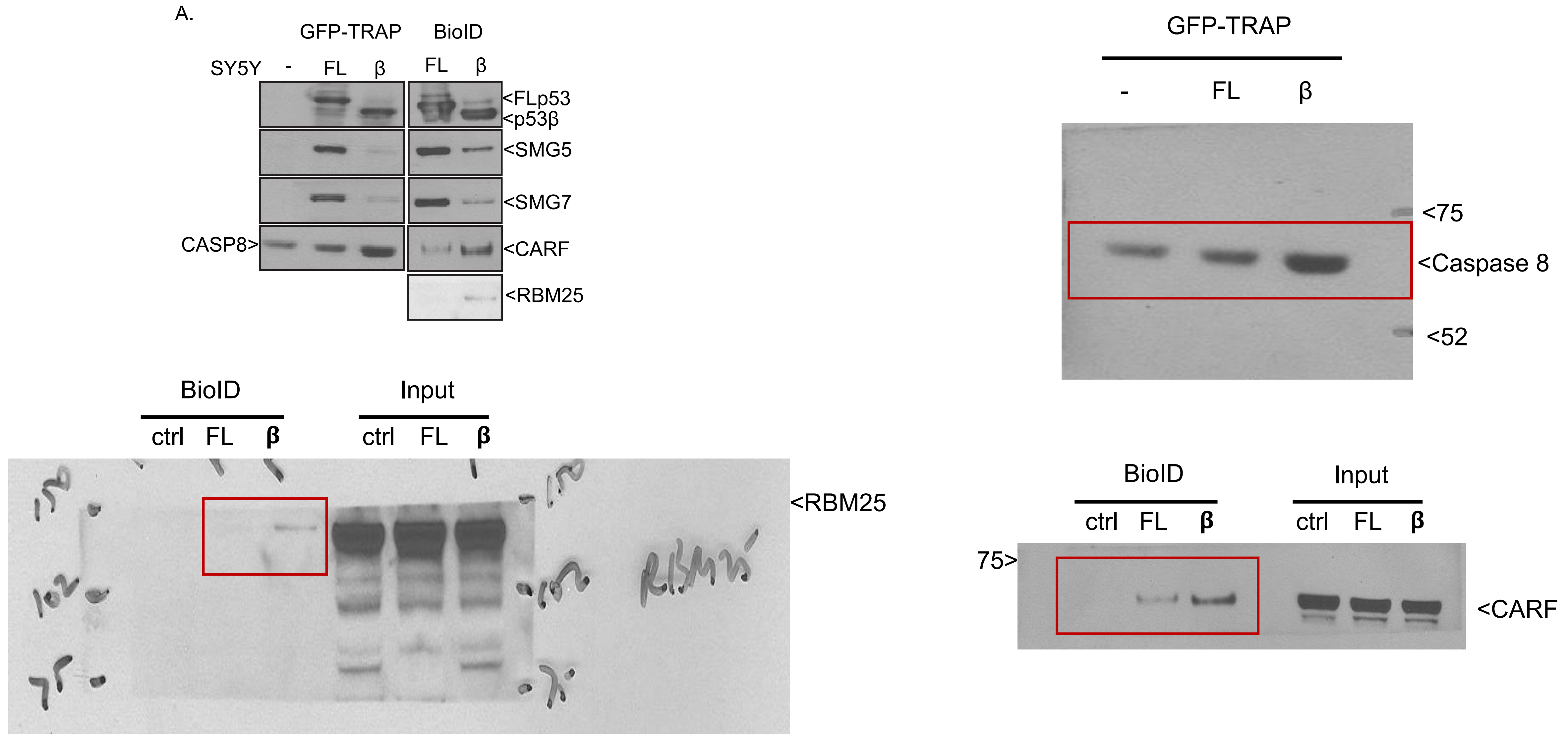
A.
GFP-TRAP
BioID
SY5Y
-
FL
β
FL
β
<FLp53
<p53β
<SMG5
<SMG7
CASP8>
<CARF
<RBM25
GFP-TRAP
-
FL
β
<75
<Caspase 8
<52
BioID
Input
ctrl
FL
β
ctrl
FL
β
<RBM25
BioID
Input
ctrl
FL
β
ctrl
FL
β
75>
<CARF
